# Correlation between Neutrophil-to-Lymphocyte Ratio and Pretreatment Magnetic Resonance Imaging and Their Predictive Significance in Cervical Carcinoma Patients Referred for Radiotherapy

**DOI:** 10.1155/2022/3409487

**Published:** 2022-03-18

**Authors:** Chunyu Liang, Zhiyuan Xu, Xinping Shen, Kusheng Wu

**Affiliations:** ^1^Department of Medical Imaging, Radiology Center, The University of Hong Kong-Shenzhen Hospital, Shenzhen 518000, China; ^2^Department of Clinical Oncology, The University of Hong Kong-Shenzhen Hospital, Shenzhen 518000, China; ^3^Department of Preventive Medicine, Shantou University Medical College, Shantou 515041, China

## Abstract

**Objective:**

The aim of this study was to determine the correlation between neutrophil-to-lymphocyte ratio (NLR) and various tumor parameters assessed by pretreatment magnetic resonance imaging (MRI) and to evaluate their prognostic significance for cervical carcinoma treated with radiotherapy (RT).

**Methods:**

The study enrolled 78 patients with biopsy-proven squamous cell carcinoma (SCC) of primary cervical cancer (clinically staged IB2 to IVA) who were treated in the Department of Clinical Oncology of the University of Hong Kong-Shenzhen Hospital between August 2015 and May 2019. A retrospective analysis of patients with SCC was performed. Firstly, we investigated the correlations between NLR and MRI parameters. Then, univariate and multivariate analyses were performed to identify the prognostic factors for overall survival (OS) and progression-free survival (PFS). Kaplan–Meier curves were constructed for OS and PFS.

**Results:**

Higher NLR showed significant association with larger tumor diameter and parametrial invasion assessed by pretreatment MRI. Univariate analysis indicated that uterine body invasion, parametrial invasion, and NLR were associated with prognosis of cervical cancer. Multivariable analyses demonstrated that parametrial invasion and NLR higher than the cutoff were independently associated with shorter OS and PFS, whereas uterine body invasion showed a significantly unfavorable influence on OS but showed no significant effect on PFS. Using the three risk factors of NLR above cutoff, parametrial invasion, and uterine body invasion, patients were divided into three subgroups. The three-year OS rates of patients with zero risk factors, one risk factor, and two or three of these factors were 96%, 91%, and 42%, respectively (*P* < 0.001), showing a downward trend.

**Conclusions:**

Uterine body invasion, parametrial invasion, and NLR were significant prognostic factors for patients with cervical carcinoma treated with RT. These results may supplement FIGO staging to improve prognostic assessment of patients.

## 1. Introduction

Cervical cancer is the second most common malignancy and third leading cause of cancer mortality among females worldwide [1]. According to the National Comprehensive Cancer Network (NCCN) Guidelines for Cervical Cancer, radiotherapy (RT) with or without concurrent chemotherapy is regarded as the standard treatment for locally advanced cervical cancer (stage IB2 or above according to the International Federation of Gynecology and Obstetrics [FIGO] staging) [2]. FIGO staging has been adopted worldwide as a prognostic tool for patients with cervical cancer. However, FIGO staging alone is not sufficient to accurately predict outcomes, with markedly different prognoses seen among patients with similar FIGO stages, especially in cases of locally advanced disease after RT. Thus, factors predicting treatment outcome and survival in patients after RT are highly warranted, with the ultimate goal to establish the foundation for personalized treatment.

For many early-stage cervical cancer patients, radical surgery is recommended. Several postoperation pathological factors, including size and morphosis of tumor, depth of tumor invasion, and lymph node status, are used as prognostic factors. However, when the cervical cancer is locally advanced, RT is recommended, rather than radical surgery, and the postoperative pathological risk factors do not apply. Magnetic resonance imaging (MRI) is vital in assessing morphological characteristics and lymph node status prior to treatment. A good correlation is seen between pretreatment parameters measured by MRI and postoperative pathological findings [3]. The MRI parameters, including tumor size, stage, uterine body invasion, and ADC value, have been described as important prognostic factors in patients with invasive cervical carcinoma [4–6]. In the meanwhile, NLR, an index of local immune response and systemic inflammation, has also been reported as an independent prognostic factor for a variety of tumors including invasive cervical carcinoma. There have been reports of shorter OS and PFS in patients with higher NLR, revealing a negative impact of high NLR on prognosis [7, 8]. Although both the NLR and pretreatment MRI have been demonstrated to influence prognosis in patients with invasive cervical carcinoma, to the best of our knowledge, any correlation between the two has not been evaluated. In addition, FIGO staging is the main tool for prognostic evaluation in cervical carcinoma but is limited as regards its ability to predict recurrence and survival after RT. During the current study, a retrospective evaluation of the prognostic factors relating to recurrence and survival in patients with locally advanced cervical carcinoma was conducted. The aim was to evaluate any correlation between NLR and various pretreatment MRI parameters and to examine their prognostic significance for cervical carcinoma treated with RT.

## 2. Materials and Methods

### 2.1. Patients

The study enrolled 78 patients with biopsy-proven squamous cell carcinoma (SCC) of primary cervical cancer (clinically staged IB2 to IVA) who were treated in the Department of Clinical Oncology of the University of Hong Kong-Shenzhen Hospital between August 2015 and May 2019, and approval was obtained from the hospital's Ethics Committee. Patients underwent MRI of the pelvis performed before the start of treatment as part of their initial clinical examination. The patients were classified according to the 2018 FIGO staging system. The extent of tumor invasion and lymph node status were mainly based on MRI findings. Of the 78 women included in the study, 36 had FIGO stage IB2-IIB disease, and 7 had stage IIIA-IIIB disease, while 35 had stage IIIC-IVA disease. The median age was about 55 years (range, 33–78 years).

### 2.2. Treatment and Follow-Up

Out of the cohort, 13 patients received RT alone and 65 were treated with concurrent cisplatin chemotherapy (40 mg/m2 weekly for six cycles). Weekly carboplatin was used as an alternative if creatinine clearance was below 50 ml/min. All 78 patients received external beam radiation therapy (EBRT) including RapidArc or three-dimensional conformal radiotherapy (3DCRT). Prescription dose of EBRT for RapidArc was 45 Gy in 25 fractions to whole pelvis including cervix, bilateral parametrium, uterus, part of vagina, and regional lymphatics with a simultaneous integrated boost of 55.0∼57.5 Gy to pelvic or retroperitoneal metastatic lymph nodes. For 3D-CRT, two sequential phases included 45 Gy/25 Fr to pelvis for phase I and FIGO IIIB 16 Gy/8 Fr, other stage 10 Gy/5 Fr boosting to pelvic wall for phase II. All EBRT was daily, 5 Fr/week. MRI guided-brachytherapy was performed 2∼3 weeks after initiation of EBRT with high dose rate, once a week for a total of 4 times. Cumulative equivalents of >84 Gy (EQD2) for stage IB ∼ IIIA and >90 Gy (EQD2) for ≥ stage IIIB were set for cervical tumor.

After the primary treatment, patients had follow-up examinations about every 3 months for the first 2 years and every 6 months thereafter. During routine follow-ups, imaging by chest computed tomography (CT) and whole abdomen or pelvis MRI were performed annually in addition to a physical examination. Lesion biopsies were performed when tumor recurrence was suspected based on pelvic imaging or physical exam. OS was defined as the interval between initial treatment and death due to cervical carcinoma, or the final follow-up, and PFS was defined as the period from initial treatment to the diagnosis of local recurrence or distant metastasis from imaging data or to final follow-up. Patients' follow-up was maintained until death or the latest date of December 2020.

### 2.3. Definition of NLR

Baseline routine complete blood counts (CBCs) were obtained within one week prior to the start of treatment. The NLR represents the ratio of absolute neutrophil count to absolute lymphocyte count.

### 2.4. MRI Acquisition and Analysis

Pretreatment MRI was performed on a 1.5T scanner (MAGNETOM Avanto; Siemens, Erlangen, Germany) using the system's body coil. Axial T1-weighted spin echo (SE) sequences; axial, sagittal, and coronal T2-weighted fast SE images of the whole pelvis; and axial diffusion-weighted images and ADC map of the whole pelvis were acquired. Then, sagittal and axial contrast-enhanced dynamic MR images were obtained following the injection of gadolinium chelate.

MRI findings (tumor diameter, vaginal extension, uterine body invasion, parametrial invasion, lymph node involvement, and ADC value) were analyzed in consensus by two senior radiologists. Tumor diameter was expressed at its largest extent, which was the maximum diameter of the *x*-, *y*-, and *z*-axes of each case. Primary tumor reaching or exceeding the anterior or posterior fornix in the sagittal plane was interpreted as vaginal invasion. Uterine body invasion was defined as tumor over the isthmus of the uterus. Replacement of the low signal intensity rim of uterine cervix in axial T2-weighted images by tumor was interpreted as parametrial invasion [9]. Lymph nodes equal to or greater than 10 mm in minimum diameter were interpreted as lymph node involvement. The ADC value was analyzed by placing at least five circular regions of interest (ROIs) over solid portion of tumor for each patient on the ADC map image, and the minimum value of the five ROIs was adopted as tumor's ADC value.

### 2.5. Statistical Analysis

The data were analyzed with SPSS 26.0 (SPSS Inc., Chicago, IL) and GraphPad Prism software (Version 9.0.0, GraphPad Prism, Inc., San Diego, CA, USA). *P* < 0.05 was determined to be statistically significant. According to the statistical power calculation by PASS software, the power value was less than 0.8.

The NLR was divided into two groups (high vs. low) according to cutoff value evaluated by receiver operating characteristic (ROC) curve. Statistical comparisons of pretreatment MRI parameters between low group and high group were made using the Student's *t*-test for continuous variables or using the chi-square test for classified variables. Relationships between NLR and MRI parameters were examined using Spearman's correlation and chi-square tests.

OS and PFS were the major endpoints of this study. The effect of pretreatment MRI parameters (tumor diameter, vaginal extension, uterine body invasion, parametrial invasion, lymph node involvement, and ADC value), routine CBCs examinations (including absolute leukocyte, neutrophil, lymphocyte, and NLR), and other factors (age and FIGO stage) on OS and PFS was investigated using the Kaplan–Meier method by log-rank test. Variables that were significant on univariate analysis (*P* ≤ 0.05) were subjected to multivariate analysis with the Cox proportional hazards model to evaluate independent factors contributing to OS and FPS. Survival curves were depicted in GraphPad Prism software with the Kaplan–Meier method and were compared with the Mantel–Haenszel log-rank test.

## 3. Results

### 3.1. Clinical Characteristics and Treatment Outcomes

**Figure 1:**
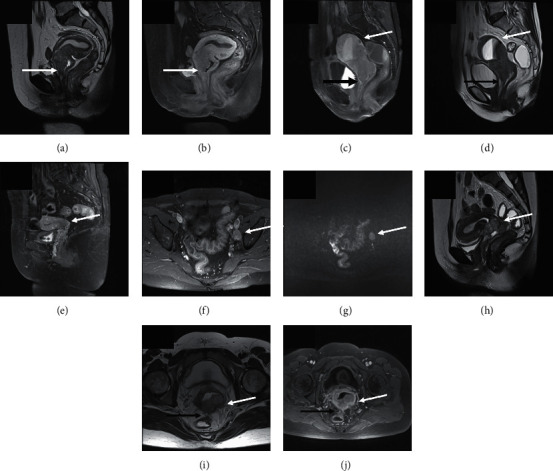
(a, b) A 53-year-old woman with IIA cervical cancer: T2-weighted sagittal image (a) and T1-weighted contrast-enhanced sagittal image (b) show a cervical mass with infiltration of the upper vagina (white arrow). (c, d) A 60-year-old woman with IIA cervical cancer: T1-weighted contrast-enhanced sagittal image (c) and T2-weighted sagittal image (d) show a bulky cervical mass with vaginal extension (black arrow) as well as uterine body invasion (white arrow). (e–g) A 67-year-old woman with IIIC cervical cancer: T2-weighted sagittal image (e) shows a cervical mass (white arrow); axial contrast-enhanced image (f) and diffusion-weighted image (g) show an enlarged pelvic lymph node (white arrow) with moderate enhancement and diffusion restriction, indicating pelvic lymph node involvement. (h–j) A 49-year-old woman with IVA cervical cancer: T2-weighted sagittal image (h) shows a cervical mass (white arrow); axial T2-weighted image (i) and contrast-enhanced image (j) show the cervix tumor (white arrow) with rectal wall invasion (black arrow).

Seventy-eight patients diagnosed and treated for cervical cancer (IB2 to IVA) were included. Mean patient age was 55.10 ± 12.27 years (range, 33–78 years). Clinical details of these cases are given in Table 1. MRI details of several cases are given in Figure 1.

Mean follow-up time was 32.5 (range, 20–65) months. The mean time of OS and PFS was 32.8 and 30.8 months, respectively. Three-year OS and PFS rates were 85.0% and 79.0%, respectively. During the period of follow-up, 14 (17.9%) developed locoregional failure or distant metastasis (liver, lung, mediastinum, or left supraclavicular lymph node metastasis). Among these, six patients developed locoregional failure, six patients had distant metastasis, and two patients presented both locoregional failure and distant metastasis. By the time of last follow-up, 13 (16.7%) cases were dead.

### 3.2. Correlation between NLR and Pretreatment MRI Parameters

**Figure 2:**
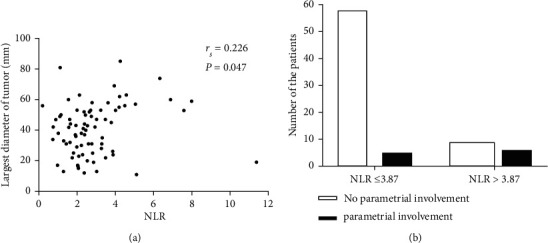
Correlations between NLR and pretreatment MRI parameters. (a) Plots of NLR against largest tumor diameter (*r* = 0.226, *P* < 0.05). Points represent single patients. (b) Association between NLR and parametrial invasion. Each bar represents the number of patients. Parametrial invasion was exhibited in 7.9 and 40.0% of patients in low (NLR ≤ 3.87) and high (NLR > 3.87) NLR groups (*χ*^2^ = 10.282, *P*=0.001). NLR was strongly correlated with parametrial invasion (chi-square test, *r* = 0.363, *P*=0.001).

ROC curve analysis was used to determine the NLR cutoff value for predicting the prognosis. The analysis identified cutoff value of 3.87 for the OS (AUC = 0.717, sensitivity 53.8%, specificity 87.7%, *P*=0.001). Then, the patients were classified into high group (NLR > 3.87) and low group (NLR ≤ 3.87) based on the cutoff value.  Table 2 shows comparative analysis of two groups. Patients of the high NLR group (NLR > 3.87) demonstrated larger tumor diameter than those of the low NLR group (47.5 mm vs. 37.7 mm, *P*=0.001), with significant relationship (Spearman's correlation, rs = 0.226, *P*=0.047). Among the patients in the low NLR group, 7.9% showed parametrial invasion, while 40.0% of patients in the high NLR group exhibited this phenomenon (*χ*^2^ = 10.282, *P*=0.001). NLR was strongly correlated with parametrial invasion (chi-square test, *r* = 0.363, *P*=0.001) (Figure 2). There was no other significant correlation between NLR and pretreatment MRI parameters.

### 3.3. Univariate Survival Analyses and Multivariate Analyses

**Figure 3:**
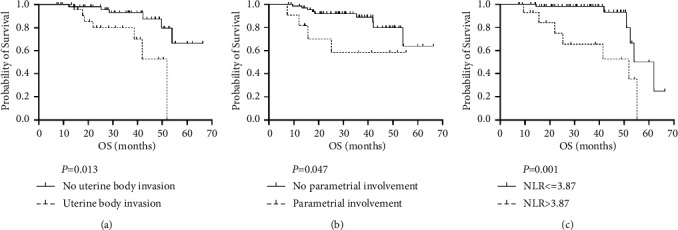
Kaplan–Meier survival plots in overall survival (OS) stratified by uterine body invasion (a), parametrial involvement (b), and NLR (c).

**Figure 4:**
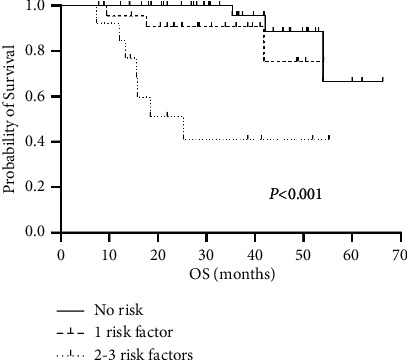
Overall survival curves of the groups divided by the number of risk factors, showing a downward trend of OS when the number of risk factors increased.

Table 3 shows the univariate analysis for OS and PFS. ROC curve analysis determined the threshold (cut-point) of ADC for OS positivity, while other continuous variables such as age, tumor diameter, and leukocyte count were translated into classified variables according to cutoff points in clinical application. Univariate analysis indicated that uterine body invasion, parametrial invasion, and NLR were associated prognosis of cervical cancer.  Figure 3 shows Kaplan–Meier curves for OS by uterine body invasion, parametrial invasion, and NLR. Parametrial invasion and an NLR above the cutoff were independently associated with shorter OS and PFS, as shown by multivariate Cox analysis. Uterine body invasion showed an adverse effect on OS but no significant influence on PFS (Table 4). No other factor could be correlated with PFS or OS. Based on the three risk factors (NLR > 3.87, parametrial invasion, and uterine body invasion) for OS, patients were assigned to one of three groups. The first group had no risk factor, the second had one risk factor, and the third had two or three risk factors. The three-year OS rates were 96% for group 1, 91% for group 2, and 42% for group 3, showing the adverse effect of the risk factors on survival. The differences in OS among these three groups were statistically significant (*P* < 0.001; Figure 4).

## 4. Discussion

The current study demonstrates that information derived from NLR and MRI complements FIGO staging in determining the prognosis of cervical cancer cases. MRI can visualize the local morphological characteristics of cervical cancer, including size, shape, and range of invasion. However, the tumor not only involves the local area, but also causes systemic immune response. NLR has been reported as an index of systemic immune response for a variety of tumors including invasive cervical carcinoma [10]. To the best of our knowledge, no previous studies have assessed the correlation between NLR and MRI in cervical cancer or other malignancies. In this study, the association between NLR and MRI was first noticed, by observing that higher pretreatment NLR was associated with larger tumor diameter and parametrial invasion assessed by MRI. Evaluating the correlation between NLR and various pretreatment MRI parameters is helpful for further understanding the interaction between local mass and host's adaptive immune system and finally provides basis for personalized treatment of patients.

In recent years, the influence of the tumor microenvironment (TME) has attracted increasing attention. Cervical carcinomas develop an abnormal TME characterized by interstitial hypertension, intratumoral hypoxia, elevated amounts of lactate, nitric oxide, arginase, vascular endothelial growth factor (VEGF), reactive oxygen (ROS), low glucose concentrations, and energy deprivation, resulting in tumor neovascularization [11, 12]. A variety of inflammatory cells and inflammatory mediators are recruited to form the TME. The peripheral leukocyte, neutrophil, lymphocyte, and platelet counts have been considered as indexes reflecting the local immune response and systemic inflammatory response to tumor. Although there are many inflammatory biomarkers, NLR has been reported as a particularly appropriate gauge of tumor-associated inflammation. In this study, we observed that higher NLR was associated with larger tumor diameter and parametrial invasion. The mechanism underlying elevated NLR and cancer progression remains difficult since the neutrophil population have been divided into protumor and antitumor phenotypes. Based on the reported literature, possible candidate mechanisms include inhibiting the immune system, suppressing the activity of lymphocytes and T-cell response, providing angiogenesis, epithelial and stromal growth factors, survival factors, protumorigenic factors, producing lytic enzymes and ROS, and developing the process of epithelial-mesenchymal transition [13, 14].

Moreover, in this evaluation, we also investigated the prognostic factors predicting locoregional recurrence and survival in cervical cancer after RT. Our results suggested that two MRI parameters (uterine body invasion and parametrial invasion) and NLR were independent predictors for local control of disease and survival. Multivariable analyses demonstrated that parametrial invasion and NLR higher than the cutoff were independently associated with shorter OS and PFS, whereas uterine body invasion exhibited a significantly negative effect on OS but showed no significant effect on PFS. Age; FIGO stage; other MRI parameters (tumor diameter, vaginal extension, lymph node involvement, and ADC value); and CBCs examinations including absolute leukocyte, neutrophil, and lymphocyte were not related to OS and PFS.

In recent years, there are many studies regarding prognostic significance of MRI for cervical cancer patients treated with RT. Some previous studies have reported that uterine body invasion accessed by MRI was associated with worse survival, which is consistent with our report. For example, Narayan et al. [15] reported that uterine body invasion was significant factor in predicting nodal involvement. FDG-PET indicated pelvic node positivity in 75% patients with uterine body invasion compared with 11% without (*P* < 0.001). Likewise, Kim et al. [5] conducted a retrospective study to evaluate the predictive value of pretreatment MRI for survival in women treated for advanced cervical cancer. Using multivariate analyses of OS, tumor size, and uterine body involvement by tumor showed a significantly unfavorable influence on OS. We are currently unaware of any reports regarding the impact of parametrial invasion on OS and PFS. It may be mainly because parametrial invasion has not previously received much attention and was not listed as a potential predictive factor in many previous studies. As we know, parametrial invasion is a considerable factor that influences FIGO staging and treatment options [16]. Association between parametrial involvement and lymph node involvement has been reported in the literature [17, 18]. Therefore, the predictive value of parametrial involvement for cervical carcinoma deserves further study. Several MRI parameters, such as tumor size, ADC value, FIGO staging, and pelvic node metastasis, which did not appear to be meaningful prognostic factors in the current study, have previously been reported to be independently associated with OS and PFS [6, 19]. One factor leading to discrepancies in the reports may be the composition of the experimental population. The cohort of the current study was mainly composed of stage IB-IIB patients (36/78; 46.2%), and clinicopathological characteristics in our study may differ from those considered by other authors. Moreover, the patient cohort was relatively small with a short follow-up time. A larger cohort with longer follow-up would make the study more reliable.

The current finding that an NLR value above the cutoff was independently associated with shorter OS and PFS is consistent with those of previous studies. Higher NLR is associated with poorer survival in patients diagnosed with cervical SCC, as well as several other types of cancer [20]. Mizunuma et al. [21] retrospectively analyzed 56 patients diagnosed with cervical SCC who were treated with RT. OS and PFS were significantly shorter in patients with a higher NLR, confirming the prognostic value of this ratio. Furthermore, patients with a lower NLR were more likely to show a complete response to treatment.

Some weaknesses are present in the current study. The retrospective nature of the investigation is the largest limitation. Moreover, just as mentioned before, the patient cohort was relatively small with short follow-up time. According to the statistical power calculation by PASS software, the power value was less than 0.8. A larger cohort with longer follow-up would make the study more reliable. In addition, the number of variables used for the multivariate model was limited. Some previous studies have recognized pretreatment squamous cell carcinoma antigen (SCC-Ag) level and human papilloma virus (HPV) load as independent prognostic factors for cervical SCC [22, 23]. HPV load and SCC-Ag level were not used as variables in the present study. A nomogram with an expanding size of study cohort, a useful tool for assessment of the individual probability of a certain event, may be another promising approach with prognostic value [24, 25]. Validation of the current findings regarding prognostic factors warrants further investigation.

## 5. Conclusions

The current analysis suggests that higher NLR showed significant association with larger tumor diameter and parametrial invasion, which were identified by pretreatment MRI. Uterine body invasion, parametrial invasion, and higher NLR were all associated with poor prognosis of cervical cancer. These findings supplement FIGO staging to provide more precise prognoses for cervical carcinoma treated with RT. Using MRI and CBCs, accurate and useful pretreatment information could be obtained, which would be advantageous and meaningful for personalization of treatment.

## Figures and Tables

**Table 1:** Patients' characteristics.

Variables	*n* (%), or mean (range)
Number of patients	78
Age (y)	55 (33–78)
BMI	23 (18–30)
NLR	2.9 (0.2–11.4)
Largest diameter of the tumor (mm)	40 (11–85)
ADC value (×10^−3^ mm^2^/s range)	0.776 (0.506–1.266)
*FIGO stage (%)*	
IB-IIB	36 (46.2)
IIIA-IIIB	7 (9.0)
IIIC-IVA	35 (44.9)
*Vagina invasion (%)*	
Negative	18 (23.1)
Positive	60 (76.9)
*Uterine body invasion (%)*	
Negative	53 (67.9)
Positive	25 (32.1)
*Parametrial involvement*	
Negative	67 (85.9)
Positive	11 (14.1)
*Pelvic lymph node metastasis*	
Negative	48 (61.5)
Positive	30 (38.5)
*Treatment (%)*	
RT alone	13 (16.7)
CCRT	65 (83.3)

BMI: body mass index; ADC: apparent diffusion coefficient; FIGO: International Federation of Gynecology and Obstetrics; NLR: neutrophil/lymphocyte ratio; RT: radiation therapy; CCRT: concurrent chemoradiation therapy.

**Table 2:** Comparison of pretreatment MRI parameters between low and high NLR in primary cervical cancer.

Variable	Low NLR group (≤3.87) (*n* = 63)	High NLR group (>3.87) (*n* = 15)	*Pvalue*
Largest diameter of the tumor, mm	37.56 ± 14.30	53.33 ± 20.31	0.001^※※^
ADC value, ×10^−3^ mm^2^/s	0.77 ± 0.15	0.79 ± 0.16	0.526
*Vagina invasion, n (%)*			
Negative	15 (23.8)	3 (20.0)	0.753
Positive	48 (76.2)	12 (80.0)	
*Uterine body invasion, n (%)*			
Negative	45 (71.4)	8 (53.3)	0.177
Positive	18 (28.6)	7 (46.7)	
*Parametrial involvement, n (%)*			
Negative	58 (92.1)	9 (60.0)	0.001^※※^
Positive	5 (7.9)	6 (40.0)	
*Pelvic lymph node metastasis, n (%)*			
Negative	46 (73.0)	10 (66.7)	0.623
Positive	17 (27.0)	5 (33.3)	

ADC: apparent diffusion coefficient; NLR: neutrophil-to-lymphocyte ratio. ^※※^*P* < 0.01.

**Table 3:** Univariate analysis for overall survival (OS) and progression-free survival (PFS).

Variable	*n*	OS (months)		*P* value	PFS (months)		*P* value
		Mean (SE)	95% CI		Mean (SE)	95% CI	
*Age*							
≤50 years	34	46.42 (3.05)	40.44–52.40	0.451	42.22 (3.45)	35.45–48.98	0.253
>50 years	44	57.04 (3.16)	50.84–63.24		54.75 (3.38)	48.12–61.38	

*FIGO stage*							
IB-IIB	36	53.98 (3.78)	46.58–61.38	0.279	47.02 (4.41)	38.39–55.66	0.109
IIIA-IIIB	7	48.89 (6.65)	35.85–61.93		40.75 (4.88)	31.19–50.30	
IIIC-IVA	35	55.59 (2.42)	50.85–60.32		54.05 (2.77)	48.63–59.47	

*Tumor diameter*							
<20 mm	9	50.20 (6.85)	36.77–63.63	0.676	45.75 (6.27)	33.46–58.03	0.840
≥20 mm, < 40 mm	28	58.59 (4.16)	50.43–66.74		52.19 (4.95)	42.49–61.89	
≥40 mm	41	50.63 (2.90)	44.94–56.32		49.01 (3.23)	42.68–55.34	

*Pelvic lymph node metastasis*							
Negative	56	54.54 (3.09)	48.48–60.60	0.496	50.25 (3.27)	43.84–56.66	0.177
Positive	22	50.02 (2.79)	44.55–55.48		49.91 (2.87)	44.28–55.54	

*Vagina invasion*							
Negative	18	55.51 (2.77)	50.69–60.94	0.316	53.21 (3.34)	46.66–59.76	0.308
Positive	60	54.26 (3.33)	47.73–60.78		51.06 (3.33)	44.54–57.58	

*Uterine body invasion*							
Negative	53	59.16 (2.67)	53.93–64.38	0.013^※^	54.26 (3.10)	48.19–60.34	0.253
Positive	25	40.49 (3.53)	33.56–47.41		39.50 (3.89)	31.88–47.11	

*Parametrial invasion*							
Negative	67	57.07 (2.82)	51.54–62.59	0.047^※^	54.24 (2.87)	48.62–59.87	0.042^※^
Positive	11	38.82 (6.43)	26.21–51.42		34.26 (6.96)	20.63–47.90	

*ADC value*							
≤0.78 × 10^−3^ mm^2^/s	39	51.50 (3.28)	45.08–57.93	0.426	48.29 (3.72)	41.01–55.57	0.639
>0.78 × 10^−3^ mm^2^/s	39	54.89 (4.34)	46.38–63.40		51.74 (4.04)	43.83–59.65	

*Leukocyte count*							
<4000 n/*μ*l	11	32.23 (3.26)	25.84–38.62	0.452	25.39 (4.37)	16.82–33.95	0.067
4000–10000 n/*μ*l	57	55.86 (3.44)	49.12–62.60		54.53 (3.04)	48.57–60.48	
>10000 n/*μ*l	10	47.13 (7.21)	33.00–61.26		46.35 (7.58)	31.49–61.21	

*Neutrophil count*							
<2000 n/*μ*l	8	33.98 (3.20)	27.72–40.24	0.388	23.61 (3.69)	16.39–30.83	0.442
2000–7000 n/*μ*l	59	55.85 (3.44)	49.11–62.59		53.72 (3.07)	47.70–59.74	
>7000 n/*μ*l	11	44.52 (7.01)	30.78–58.26		43.81 (7.31)	29.49–58.13	

*Lymphocyte count*							
<1500 n/*μ*l	25	39.30 (2.68)	34.04–44.56	0.219	35.76 (3.56)	28.79–42.74	0.050
1500–2500 n/*μ*l	42	54.25 (2.40)	49.53–58.96		51.68 (2.86)	46.07–57.29	
>2500 n/*μ*l	11	57.62 (5.30)	47.23–68.01		57.16 (5.75)	45.88–68.43	

*NLR*							
≤3.87	63	58.51 (2.96)		0.001^※※^	55.88 (2.85)	50.30–61.46	0.002^※※^
>3.87	15	55.63 (2.67)			31.32 (5.86)	19.84–42.79	

CI: confidence interval; OS: overall survival; PFS: progression-free survival; ADC: apparent diffusion coefficient; FIGO: International Federation of Gynecology and Obstetrics; NLR: neutrophil-to-lymphocyte ratio. ^※^*P* < 0.05; ^※※^*P* < 0.01.

**Table 4:** Multivariate analysis of factors according to overall survival (OS) and progression-free survival (PFS).

Factor	OS			PFS		
	HR	95% CI	*P* value	HR	95% CI	*P* value
Uterine body invasion	3.726	1.152–12.053	0.028^※^	1.845	0.694–4.904	0.220
Parametrial involvement	4.112	1.212–13.957	0.023^※^	3.581	1.214–10.562	0.021^※^
NLR	3.120	1.202–8.100	0.019^※^	3.216	1.193–8.671	0.021^※^

HR: hazard ratio; CI: confidence interval; OS: overall survival; PFS: progression-free survival; NLR: neutrophil-to-lymphocyte ratio. ^※^*P* < 0.05.

## Data Availability

The data used to support the findings of this study are available from the corresponding author upon request.
